# A Simplified Cluster Analysis of Electron Track Structure for Estimating Complex DNA Damage Yields

**DOI:** 10.3390/ijms21051701

**Published:** 2020-03-02

**Authors:** Yusuke Matsuya, Toshiaki Nakano, Takeshi Kai, Naoya Shikazono, Ken Akamatsu, Yuji Yoshii, Tatsuhiko Sato

**Affiliations:** 1 Nuclear Science and Engineering Center, Research Group for Radiation Transport Analysis, Japan Atomic Energy Agency, 2-4 Shirakata, Tokai, Ibaraki 319-1195, Japan; 2 Department of Quantum life Science, Quantum Beam Science Research Directorate, National Institutes of Quantum and Radiological Science and Technology, 8-1-7 Umemidai, Kizugawa-shi, Kyoto 619-0215, Japan; 3 Central Institute of Isotope Science, Hokkaido University, Kita-15 Nishi-7, Kita-ku, Sapporo, Hokkaido 060-0815, Japan

**Keywords:** Monte Carlo radiation transport, complex DNA damage coupled with base damage, modelling of DNA damage yields

## Abstract

Complex DNA damage, defined as at least two vicinal lesions within 10–20 base pairs (bp), induced after exposure to ionizing radiation, is recognized as fatal damage to human tissue. Due to the difficulty of directly measuring the aggregation of DNA damage at the nano-meter scale, many cluster analyses of inelastic interactions based on Monte Carlo simulation for radiation track structure in liquid water have been conducted to evaluate DNA damage. Meanwhile, the experimental technique to detect complex DNA damage has evolved in recent decades, so both approaches with simulation and experiment get used for investigating complex DNA damage. During this study, we propose a simplified cluster analysis of ionization and electronic excitation events within 10 bp based on track structure for estimating complex DNA damage yields for electron and X-ray irradiations. We then compare the computational results with the experimental complex DNA damage coupled with base damage (BD) measured by enzymatic cleavage and atomic force microscopy (AFM). The computational results agree well with experimental fractions of complex damage yields, i.e., single and double strand breaks (SSBs, DSBs) and complex BD, when the yield ratio of BD/SSB is assumed to be 1.3. Considering the comparison of complex DSB yields, i.e., DSB + BD and DSB + 2BD, between simulation and experimental data, we find that the aggregation degree of the events along electron tracks reflects the complexity of induced DNA damage, showing 43.5% of DSB induced after 70 kVp X-ray irradiation can be classified as a complex form coupled with BD. The present simulation enables us to quantify the type of complex damage which cannot be measured through *in vitro* experiments and helps us to interpret the experimental detection efficiency for complex BD measured by AFM. This simple model for estimating complex DNA damage yields contributes to the precise understanding of the DNA damage complexity induced after X-ray and electron irradiations.

## 1. Introduction

Ionizing radiation within the human body causes DNA damage [1] both physically (i.e., energy deposition) [2,3] and chemically (i.e., free radical) reacting to the DNA target [4,5,6,7]. Among various DNA damage types [8,9], DNA double-strand breaks (DSBs), defined as two strand breaks within 10 base pairs (bp) [2,10,11] are conventionally recognized as fatal DNA damage, which can lead to cell death with a certain probability [12]. Therefore, the relative biological effectiveness (RBE) at the endpoints of DSBs and cell survival has been investigated *in vitro* and *in silico* in previous reports [2,6,8,9,12,13,14,15,16,17,18,19]. Additionally, complex DNA damage composed of at least three vicinal lesions caused within 10–20 bp, such as DSBs coupled with strand breaks or base damage (BD), is believed to be more lethal to cells than simple DSBs [20,21] due to refractory damage [22,23]. To assess lethality, it is necessary to quantify the clustering degree of DNA damage with both experiments [24,25] and simulations [8,26,27,28,29]. It has been pointed out that a considerable amount of complex DNA damage can be induced after irradiation; however, the yield and nature of complex DNA damage are still difficult to be experimentally measured [30]. Due to the difficulty of measuring complex DNA damage, the validity of simulations has not been sufficiently demonstrated yet.

Experimental methods for detecting complex DNA damage have evolved in recent decades [24,30,31,32,33,34,35,36]. Among several techniques [24,30,31,32,33,34,35,36], microscopic operations coupled with an antibody against γ-H2AX [31,32,33,34] enables researchers to obtain spatial distributions of DSB induction in the cell nucleus. The adjacent degree of the DSB site can be evaluated from γ-H2AX foci volume in an assay [35,36]; however, the damage complexity at the nano-meter scale (the scale of DNA) cannot be obtained due to the limited spatial resolution from hundreds of nm to a few μm [35,36]. Meanwhile, complex DNA damage composed of BD can be quantified by means of gel electrophoresis after treatment of base excision repair enzymes [24,30,37,38] and fluorescence resonance energy transfer [39,40]. The structure of complex DNA damage, i.e., the number of lesions per damaged site was recently revealed with atomic force microscopy (AFM), where an individual BD in a complex damage site is specifically labelled with biotin/avidin coupled with an aldehyde reactive probe (ARP) [41]. However, whether all complex BD caused within a few bp can be detected in the AFM experiment [41] or not remains unknown. Thus, it is essential to compare simulations based on track structure with the corresponding experimental data, which contributes to interpreting the detection efficiency of complex BD.

A cluster analysis of inelastic interactions based on a radiation track structure in liquid water has been conducted as a powerful tool for estimating DNA damage yield [2,3,8,16,42]. The aim of this study is to develop a simple model for estimating complex DNA damage (i.e., isolated DSB, DSB + BD, DSB + 2BD, 2BD, 3BD, 4BD) based on previous simulation techniques [17,27,28], and to investigate the simulation accuracy and the nature of X-ray- (and electron-) induced complex damage in comparison with experimental data. This work finally quantifies the complexity of DNA damage under X-ray and electron irradiations.

## 2. Results and Discussions

### 2.1. Comparison between the Present Model and Experimental Complex Damage

**Figure 1:**
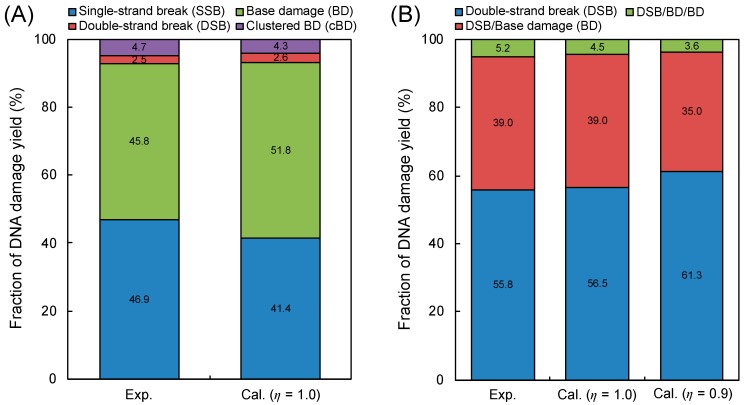
Model validity in comparison with experimental data. (**A**) is the fractions of isolated damage (single-strand break (SSB), base damage (BD)) and complex damage (double-strand break (DSB), complex BD (cBD)); (**B**) is the fractions of simple DSB, DSB/BD and DSB/BD/BD. The experimental data were obtained from the literature with treatment of base excision repair enzymes after 150 kVp X-ray irradiation [37,38] for (**A**) and with the direct observation technique with AFM after 70 kVp X-ray irradiation [41] for (**B**). The calculation represents the present model estimation based on Equations (2)–(5) and cluster analysis based on Table 1, and the determination of isolated or complex damage also follows the summary in Table 1.

We first checked the model validity, in detail yield ratio of base damage (BD) and single-strand break (SSB) = 1.3, comparing the simulation results (Equations (2)–(5) defined in Materials and Methods) with experimental data on yield fractions of SSB, double-strand beak (DSB), BD and complex BD (cBD) measured by enzymatic cleavage [37,38]. Figure 1A shows the fractions of SSB, DSB, BD and cBD obtained from our model and experiments [37,38]. Considering the good agreement between the estimation (6.9% total complex damage, 2.6% for DSB and 4.3% for cBD) and experimental data (7.2% total complex damage, 2.5% for DSB and 4.7% for cBD) in Figure 1, the developed model for complex BD considering BD/SSB = 1.3 seems reasonable. Additionally, the assumed induction ratio of BD/SSB = 1.3 could be further validated from the cross sections for impact to the DNA strand and base presented by Bernhardt et al. [43].

To directly compare our simulation results with experimental complex damage data [41], we considered the experimental detection efficiency *η* in our simulation. Focusing on the type of complex damage, as shown in Figure 1B, the present cluster analysis for identifying complex DSB types, in which the efficiency *η* = 0.9 was considered, also accurately reproduced the experimental results [41]. It should be noted that the yields of a BD and a cBD, *Y*_BD_ and *Y*_cBD_, are proportional to the efficiency *η* and the square *η*^2^, respectively (e.g., *Y*_BD_ × *η* and *Y*_cBD_ × *η*^2^). Regarding the case of *η* = 1.0, the simulation was in better agreement with the experimental data [41] than the estimated value with *η* = 0.9, proving that the efficiency for detecting BD under atomic force microscopy (AFM) operation is over 90% which is consistent with the experimental efficiency [41]. Moreover, this agreement shown in Figure 1B suggests (i) that the density of ionizations and electronic excitation events clearly reflects the damage complexity, (ii) that inter-lesion distance in the AFM experiment is within approximately 10 bp and (iii) that 9 ionization and electronic excitation events are needed for inducing one additional BD at the DSB site.

Based on the comparison results shown in Figure 1, the simple methodology in the present DNA damage model is reasonable and sufficient for estimating complex BDs and for identifying the number of BDs at the DSB site. This indicates that the cluster analysis techniques [44,45] also are reasonable for predicting complex DNA damage.

### 2.2. Testing for Consistency with Other Simulations by Different Codes

We determined the complex damage type (i.e., double-strand break (DSB), DSB/base damage (BD), DSB/BD/BD) using the present model based on a simple cluster analysis using the number of events in a small volume with 10 bp radius, *N*_cl_ (based on Table 1). The additional comparison between the present model and the previous simulations, such as by Kurbuc [44] and Geant4-DNA [45], thus becomes strong evidence to prove that these assumptions of 9 events for inducing one additional BD at the DSB site and the yield ratio of BD/SSB = 1.3 is reasonable. Added to the comparison between the simulation and the experimental data [37,38,41] (as shown in Figure 1), we also tested if this cluster model can reproduce the previous simulation data on complex DSB [44,45], i.e., DSB+ and DSB++, or not.

The comparison results between the present model coupled with Particle and Heavy Ion Transport System (PHITS) (based on Table 2) and previous simulation data calculated by Kurbuc [44] and Geant4-DNA [45] are summarized in Table 3. The present model with the PHITS calculation indicates that more than 20–30% of DSBs can be classified as complex forms. Among the 0.3 keV, 1 keV, 10 keV and 100 keV electrons, the yield of DSBs in the case of the 0.3 keV electron is highest [17], while the fraction of complex DSBs (cDSBs composed of DSB+ and DSB++) for the case of 1 keV electron is highest. This comparison indicates that the present model can reproduce the simulation results by using a different prediction model from the previous simulations [44,45], suggesting that this identification approach for complex damage type using the number of events in sites with a 10 bp radius (9 and 12 events for inducing a BD and a SB at each DSB site, respectively) is reasonable. Note that the other codes provide the complex damage yield based on the events and energy deposition to the DNA cylinder [2,29,44,45] and ion cluster size [46]. Considering the good agreements with other simulations [44,45] and the experimental data [41], this simplified model with a cluster analysis is, therefore, sufficient for identifying the complexity of DNA damage induced after X-ray and electron irradiations.

### 2.3. Interpretation of Complex Base Lesions Directly Measured by Atomic Force Microscopy (AFM) Imaging

The most recent atomic force microscopy (AFM) techniques for detecting complex base damage (BD) enables us to quantify the complex damage type, i.e., BD/BD, BD/BD/BD and BD/BD/BD/BD in addition to complex double-strand breaks (DSBs) coupled with BD (e.g., DSB/BD and DSB/BD/BD) [41]. Concerning this detection technique for BD, streptavidin labelling of DNA with aldehyde reactive prob (ARP) and AFM imaging has to be used. To reproduce the experimental data on complex BD directly measured by AFM imaging [41], we must consider several experimental efficiencies, such as the enzymatic reaction efficiency and spatial resolution for ARP. Regarding this, we next estimated the fractions of complex BD and complex DSBs coupled with BD, and tried to reproduce the experimental results measured by AFM imaging [41]. This comparison between simulation and experiments contributes to the interpretation of a direct observation technique for complex BD.

**Figure 2:**
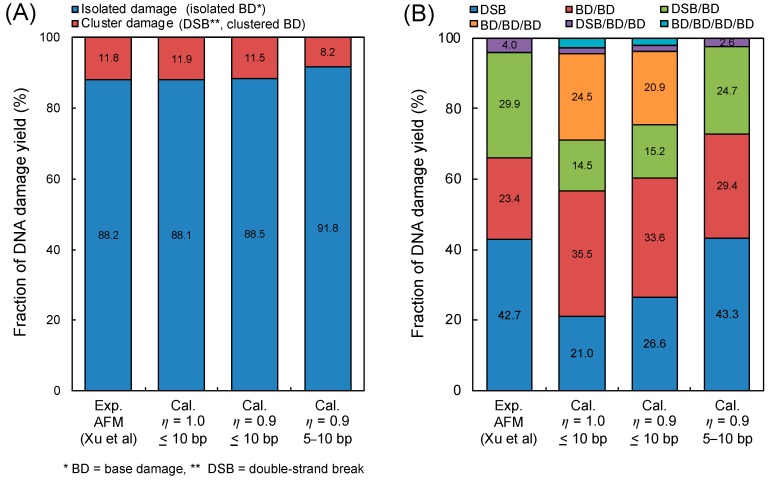
DNA damage complexity obtained by atomic force microscopy (AFM) imaging and simulation. (**A**) is the fraction of isolated base damage (BD) and complex damage (complex BD (cBD), double-strand break (DSB)); (**B**) is the type of complex DNA damage (DSB, BD/BD, DSB/BD, BD/BD/BD, DSB/BD/BD, BD/BD/BD/BD). The experimental results were obtained using AFM imaging for complex damage with BD reported by Xu et al. [41], where the energy of the X-ray is 70 kVp. The calculation represents the estimation based on Equations (2)–(5), where the determination of isolated or complex damage follows the summary in Table 1.

Figure 2 shows the comparison between the simulation results by our model and the experimental data [41], where (A) is the fraction of isolated BD and the complex damage composed of DSB and cBD, and (B) is the fractions of DSB, BD/BD, DSB/BD, BD/BD/BD, DSB/BD/BD and BD/BD/BD/BD. It should be noted that the inter-lesion distance within 10 bp was used for estimating the yield of complex damage. Regarding both cases of *η* = 1.0 and 0.9, the calculated fractions of complex damage were in good agreement with the experimental results [41], as shown in Figure 2A. Additionally, as shown in Figure 2B, highly complex forms (i.e., BD/BD/BD) and a high fraction of BD/BD can be seen in the simulation results, while the experiments did not show such tendencies.

From the comparisons in Figure 2 to reproduce the experimental results, we next considered the loss for detecting complex BD composed of two vicinal lesions caused within 5 bp due to the big ARP size (approximately a 10 bp diameter). Considering these, the yield of complex BD with the detection loss, *Y*_cBD_^*^, can be expressed as:

(1) $${Y_{\mathrm{cBD}}}^{*}=Y_{\mathrm{cBD}}\left(10\;\mathrm{bp}\right)-Y_{\mathrm{cBD}}\left(5\;\mathrm{bp}\right)$$

where *Y*_cBD_(10 bp) and *Y*_cBD_(5 bp) are the yields of complex BD caused within 10 bp and 5 bp, respectively. Under this assumption, the detection of complex BD containing more than two BDs within 10 bp was estimated to be completely impossible from the simulation standpoint. The estimations based on Equation (1) also are described as right bars in Figure 2A,B. The estimated fractions of complex damage were in good agreement with the experimental data, compared to the simulation without the detection loss. Based on these results, we found that the detection for complex BD (especially, 3BD and 4BD) was still difficult by means of an *in vitro* experiment. The experimental results for the cBD, i.e., 2BD, 3BD and 4BD, thus should be corrected using this simulation technique.

The experimental process for detecting BD in AFM [41] induced all types of BDs treated with DNA glycosylases (resulting apurinic/apyrimidinic (AP) sites) which can be labelled with an ARP that has both the alkoxyamine for the reaction with the aldehyde group of DNA and the biotin moiety for the subsequent labelling. Since the biotin moiety bound to DNA can be tagged with streptavidin (53 kDa as a large molecule), the resulting ARP-streptavidin complex can be visualized with AFM. During the series of the labelling processes, it was experimentally interpreted that overlapping and uncoupling biotins might result in a reduced efficiency for visualizing complex BDs induced within a few bp, i.e., down to about 70–80% [41]. Regarding this, the evaluation shown in Figure 2 might reflect a 3D structure problem and reduced labelling efficiency.

### 2.4. Estimation of Complex DNA Damage for Mono-Energetic Electron

The model for estimating the yields of complex DNA damage was tested in these comparisons shown in Figure 1 and Table 3. Using the present model, we further calculated the yields of the complex base damage (BD) including at least two BD, *Y*_cBD_, and the ratio of the complex and isolated BD, *Y*_cBD_/*Y*_BD_, as functions of incident electron energy.

**Figure 3:**
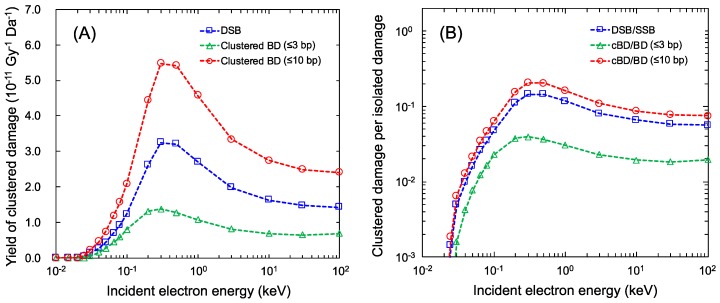
Yields of complex damage for mono-energetic electrons: (**A**) is the yields of complex base damage (BD) for various cluster sizes; (**B**) is the yield ratio of complex damage and isolated damage (complex BD (cBD) per BD and double-strand break (DSB) per single-strand break (SSB)). The blue square is *Y*_DSB_/*Y*_SSB_ for a <10 bp cluster size, the red circle is the *Y*_cBD_/*Y*_BD_ for a <10 bp cluster size, and the green triangle is the *Y*_cBD_/*Y*_BD_ for a <3 bp cluster size.

*Y*_cBD_ and *Y*_cBD_/*Y*_BD_, estimated by using the maximum inter-lesion distance *L*_c_ = 10 bp (used for comparisons) for mono-energetic electron exposure, are shown as the red circle in Figure 3. We also show *Y*_DSB_ and *Y*_DSB_/*Y*_SSB_ using the blue square in Figure 3 to compare *Y*_DSB_ with *Y*_cBD_. As reported previously [17], the peak of the number of linkages per incident electron energy was found to be around 0.3 keV electrons. The maximum *Y*_DSB_ and *Y*_cBD_ for a 10 bp cluster size (*L*_c_ = 10 bp) also were found to be 3.24 × 10^−11^ (Gy^−1^Da^−1^) (*Y*_DSB_/*Y*_SSB_ = 14.5%) and 4.58 × 10^−11^ (Gy^−1^Da^−1^) (*Y*_cBD_/*Y*_BD_ = 16.2%), respectively. The cluster size for complex BD (cBD) was conventionally set to be 3 bp, corresponding to 1.0 nm in other simulations [8]. Regarding this, we also calculated the cBD caused within 3 bp, which is shown as the green triangle in Figure 3. Changing the cluster size down to 3 bp, the maximum *Y*_cBD_ becomes much lower than that with *L*_c_ = 10 bp (*Y*_cBD_ = 1.37 × 10^−11^ (Gy^−1^Da^−1^), *Y*_cBD_/*Y*_BD_ = 3.96% for 0.3 keV electron).

**Figure 4:**
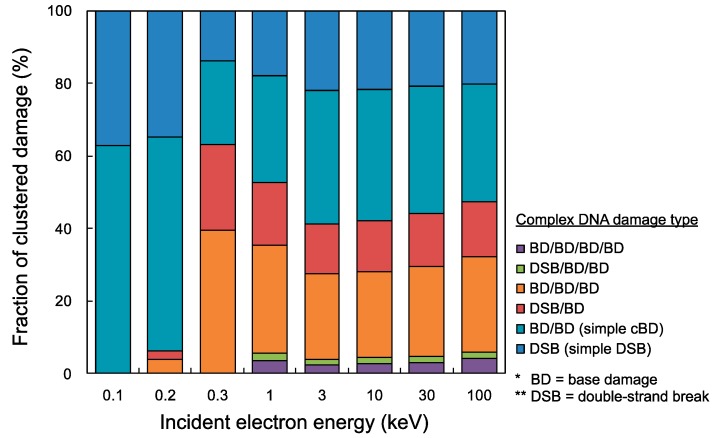
Fractions of cluster DNA damage yields for mono-energetic electron. The blue bar graph is simple double-strand break (DSB), light blue is simple complex base damage (cBD) as BD/BD, red is DSB/BD, orange is BD/BD/BD, green is DSB/BD/BD, and purple is BD/BD/BD/BD. It should be noted that the maximum inter-lesion distance and detection efficiency *η* were set as 10 bp and 1.0, respectively.

Using the present cluster analysis for predicting damage complexity, we finally estimated yield fractions of all complex DNA damage types (e.g., DSB, BD/BD, DSB/BD, BD/BD/BD, DSB/BD/BD, BD/BD/BD/BD). Figure 4 shows the fractions of complex DNA damage yields, i.e., simple DSB, BD/BD, DSB/BD, BD/BD/BD, DSB/BD/BD, BD/BD/BD/BD. Simple DSB and simple cBD (BD/BD) can be caused in a wide electron energy range of 100 eV–100 keV (shown as the blue and the light blue bar graph), while complex damage including more than two lesions cannot be induced in low energy electrons with 0.1 keV (shown as red, orange, green and purple in the bar graph). The integrated fractions of complex DNA damage including at least three lesions (e.g., DSB/BD, BD/BD/BD, DSB/BD/BD, BD/BD/BD/BD) for 0.3 keV electrons were the highest among the mono-energetic electrons below 100 keV, which was 63.1%. However, complex damage including four lesions (DSB/BD/BD, BD/BD/BD/BD) was induced by higher energy electrons than 0.3 keV, and the yield fraction for 1–100 keV electrons was 5.1% on average.

## 3. Materials and Methods

### 3.1. Monte Carlo Simulations of X-ray and Electron Processes

To compare our model with the experimental yield of complex DNA damage after X-ray irradiation [37,38,41], we used two types of spectrums of X-rays: 150 kVp [37,38] and 70 kVp [41], both with a 0.2 mm Al filter, and simulated them with the Particle and Heavy Ion Transport System (PHITS) code [47]. The X-ray spectrums were estimated according to the semiempirical model reported by Tucker et al. [48]. We adapted an electron gamma shower (EGS) [49] mode for photon transport and an electron track structure mode (etsmode) [50,51,52,53,54,55] for electron transport in the PHITS calculation. It should be noted that the “etsmode” implemented in the PHITS code was verified from various endpoints including range, stopping power, nanodosimetry and double-strand break (DSB) yield [17]. We sampled the secondary electron spectrums generated by 150 kVp and 70 kVp X-rays and transported the electrons in liquid water. The cut-off energy of electrons was set as 1 eV. The coordinates of inelastic interactions were then output using a tally named “t-userdefined”, as reported previously [17].

### 3.2. Model for Estimating Single- and Double-Strand Break Yields

Using the calculated electron spectrum, we estimated strand break yields. According to the DNA damage model previously developed [17], the number of the events, *N*_event_, and that of the linkages within 3.4 nm (10 bp), *N*_link(10)_, were sampled to calculate the yield of single-strand breaks (SSBs) *Y*_SSB_ and that of double-strand breaks (DSBs) *Y*_DSB_ in Gy^−1^Da^−1^. *Y*_SSB_ and *Y*_DSB_ can be calculated by:

(2) $$Y_{\mathrm{SSB}}\;=\;k_{\mathrm{SSB}}\;\frac{N_{\mathrm{event}}}{E_{\mathrm{dep}}},$$

(3) $$Y_{\mathrm{DSB}}\;=\;k_{\mathrm{DSB}}\;\frac{N_{\mathrm{link}(10)}}{E_{\mathrm{dep}}},$$

where *k*_SSB_ = 5.66 × 10^−12^ (keV Gy^−1^Da^−1^), *k*_DSB_ = 1.61 × 10^−13^ (keV Gy^−1^Da^−1^) [17], and *E*_dep_ is the energy deposited by electron inelastic events in keV. These coefficients of *k*_SSB_ and *k*_DSB_ were found to reproduce the experimental yields of SSB and DSB after exposure to 220 kVp X-rays in our previous report [17]. It should be noted that 10 bp was defined as the classical distance for two SSBs leading to a DSB [11]. Based on Equations (2) and (3), we calculated the DNA strand break yields under 150 kVp, 70 kVp X-rays and mono-energetic electron irradiations.

### 3.3. Model for Estimating Base Damage Yields

We obtained the coefficient of base damage (BD) induction, *k*_BD_ (keV Gy^−1^Da^−1^), in the presence of a 10 mM tris (hydroxymethyl) aminomethane-HCl buffer from the experimental literature reporting the yield ratio of BD and single-strand break (SSB), which was given as *k*_BD_/*k*_SSB_ = 1.3 [37]. It should be noted that the tris-HCl concentration was almost equivalent to liquid water due to the low radical scavenging capacity [37]. Based on this ratio, we deduced the coefficients of isolated BD and complex BD (cBD) to be *k*_BD_ = *k*_SSB_ × 1.3 = 7.36 × 10^−12^ (keV Gy^−1^Da^−1^) and *k*_cBD_ = *k*_DSB_ × 1.3^2^ = 2.72 × 10^−13^ (keV Gy^−1^Da^−1^), respectively. Using the same manner as in the strand break case, the yields of BD and cBD can be expressed by:

(4) $$Y_{\mathrm{BD}}\;=\;k_{\mathrm{BD}}\;\frac{N_{\mathrm{event}}}{E_{\mathrm{dep}}},$$

(5) $$Y_{\mathrm{cBD}}\;(L_{c})\;=\;k_{\mathrm{cBD}}\;\frac{N_{\mathrm{link}(L_{c})}}{E_{\mathrm{dep}}},$$

where *Y*_BD_ and *Y*_cBD_ are the yields of BD and cBD in Gy^−1^Da^−1^, respectively, and *L*_c_ is the maximum distance in bp between two events to sample the linkage. Because the maximum inter-lesion distance, *L*_c_, for cBD depends on the experimental detection conditions, *Y*_cBD_ should be the yield as a function of *L*_c_. Complex BD can be detected as non-DSB in the enzymatic cleavage technique [37,38], while the diameter of an aldehyde reactive prob (ARP) avidin labelled at a BD site is equal to about 1.5 times the width of a DNA ladder (2.3 nm) in the direct observation technique for BD by atomic force microscopy (AFM) [41]. Based on these, the parameter *L*_c_ was set to be 10 bp (3.4 nm) for comparing the simulation experiments with both enzymatic cleavage and AFM techniques. Considering that the distance between two adjacent ARPs can be within 10 bp, we assumed that *L*_c_ was equal to 10 bp in the simulation for the AFM experiment. Using this value for parameter *L*_c_, we calculated the yields of isolated and complex BD [37,38,41]. After comparison with the corresponding experimental data, we also estimated *Y*_cBD_ under the conditions of *L*_c_ = 3 bp as an example inducing much toxicity.

### 3.4. A Cluster Analysis for Determining Complex Damage Type

**Figure 5:**
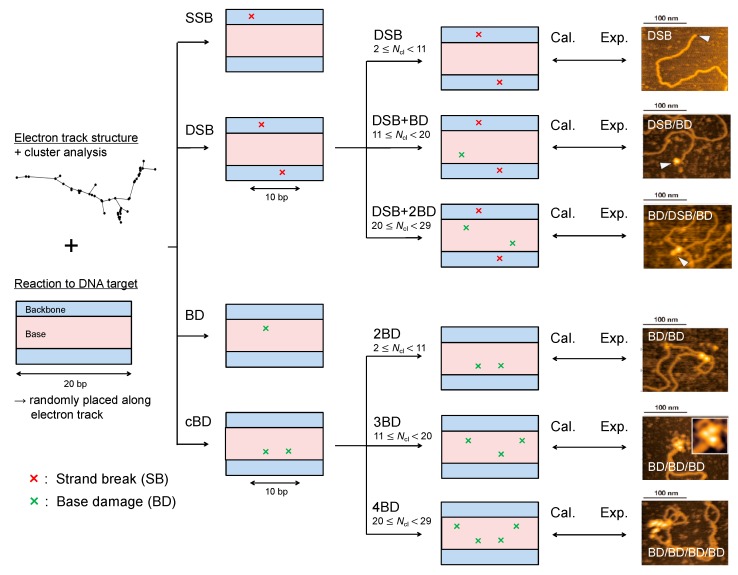
Identification of DNA damage type in the present simulation. The illustration on the left represents an electron track structure and a DNA cylinder. Considering the distance between two ionization and electronic excitation events, we identified single-strand break (SSB), double-strand break (DSB), base damage (BD), complex BD (cBD). The complexities of DSB and cBD then were determined from the number of events per cluster, *N*_cl_, at the complex damage site (DSB and cBD). After estimating the yield of each DNA damage type, we compared the estimated results with experimental data [37,38,41] (right images are example pictures obtained with AFM [41]).

Because the modelling for the yield estimation of double-strand break (DSB) and complex base damage (cBD) does not enable us to determine the damage complexity, we added a cluster analysis for calculating the event density around a DSB or cBD site. We counted the number of ionization and electronic excitation events within a sampling site with the *L*_c_ radius at a DSB or cBD site based on the number of events per cluster, *N*_cl_. Then, we determined the type of damage complexity from *N*_cl_ in reference to the cluster analysis reported by Yoshii et al. [27]. To reproduce the complex DSB coupled with base damage (BD) measured in the literature [41], it was estimated that approximately 9 events per cluster were needed on average to induce a BD within a 10 bp separation from a DSB or 2BD (cBD) site. Additionally, the mean *N*_cl_ to induce a simple DSB or 2BD site was calculated to be 6 in our previous study [17]. We therefore assumed that the ranges of *N*_cl_ to induce simple DSB (2BD), DSB+ BD (3BD) and DSB + 2BD (4BD) are 2 ≤ *N*_cl_ < 11 (6 on average), 11 ≤ *N*_cl_ < 20 (15 on average), 20 ≤ *N*_cl_ < 29 (24 on average), respectively. Under these assumptions, the mean deposition energy to cause DSB + BD within 10 bp, i.e., *N*_cl_ = 15, was estimated to be 121.7 eV, which was within the range of its reference value (102.5–122.6 eV) given by a different calculation [29]. The criteria for determining DNA damage type is summarized in Figure 5 and Table 1. Using the fraction of *N*_cl_, *f*(*N*_cl_), and the equations listed in Table 1, we calculated the yields of DNA damage, i.e., single-strand break (SSB), DSB (simple DSB), complex DSB (e.g., DSB + BD, DSB + 2BD), isolated BD, and complex BD (e.g., 2BD, 3BD, 4BD).

### 3.5. Comparison between Estimation and Experimental Data

We compared the estimated fractions of single-strand break (SSB), double-strand break (DSB), base damage (BD) and complex BD (cBD) with experimental results measured by enzymatic cleavage [37,38]. Using this comparison, we checked the model performance for estimating the yield ratios, BD/SSB and cBD/BD. Then, we compared the fractions of complex DSB (e.g., DSB, DSB/BD, DSB/BD/BD) estimated by this model with experimental data measured by the atomic force microscopy (AFM) imaging [41] to check the performance of this cluster analysis for estimating complex DSB.

After that, to interpret the most recent technique for detecting complex damage type using AFM [41], we compared the estimated fractions of isolated BD and complex damage (cBD and DSB) yields with those measured in the AFM experiment [41]. We also identified all complex damage type (e.g., DSB, BD/BD, DSB/BD, BD/BD/BD, DSB/BD/BD, BD/BD/BD/BD) shown in Figure 5 and calculated the fraction of each complex form. We then compared the estimated complex damage fractions with the experimental data [41]. It should be noted that the inter-lesion distance was set to ≤10 bp throughout this analysis.

### 3.6. Additional Benchmark Test of Cluster Analysis for Identifying Complex DSBs

Classically, the yields of complex double-strand breaks (DSBs) containing one or more strand breaks (SBs) within a 10 bp separation has been reported. These yields are designated as DSB+ (DSB coupled with one SSB) and DSB++ (DSB coupled with two strand breaks). Added to the comparison of DSB coupled with base damage (BD) between this estimation and the experimental data [37,38,41], we also compared our calculation results of DSB+ and DSB++ with the computational results in the literature [44,45]. Under the assumptions that the mean *N*_cl_ to induce an additional BD was 9 and the BD/SSB ratio was 1.3 [37], we then deduced that 12 events were needed on average for inducing an additional strand break at a DSB site. Applying the present cluster analysis and the criteria to additionally induce strand breaks (listed in Table 2), we identified the DSB+ from a DSB site on the basis of 14 ≤ *N*_cl_ < 26 and DSB++ from a DSB site on the basis of 26 ≤ *N*_cl_ < 38. Regarding the case of mono-energetic electron irradiation of 0.3 keV, 1 keV, 10 keV and 100 keV, we compared the DSB complexity (simple DSB, DSB +, DSB ++) estimated by our simulation to other simulations calculated by the Kurbuc [44] and the Geant4-DNA codes [45].

## 4. Conclusions

During this work, we compared experimental data of complex DNA damage to computational results based on an electron track structure calculated by the Particle and Heavy Ion Transport System (PHITS) code. Using the comparison between the simulations and the experimental data for complex DNA damage yields, it was confirmed (i) that the yield ratio of base damage (BD) and single-strand break (SSB) is 1.3; (ii) that the spatial pattern (density) of ionization and electronic excitation events reflects the damage complexity; and (iii) that 9 and 12 ionization and electronic excitation events are needed for inducing an additional one BD and one strand break at a double-strand break (DSB) site within a 10 bp separation. The present results indicate that conventional cluster analysis for inelastic interactions is a powerful tool and reasonable for reproducing the experimental complex DNA damage. Additionally, this model estimation can contribute to the interpretation of the experimental efficiency for detecting BD at complex damage sites and presents the fractions of complex DNA damage yields after X-ray and mono-energetic electron irradiations. While further development of this current model for high-LET irradiation is essential, this work can provide a simplified model for estimating the yield of complex DNA lesions which connects experimental and track structure simulations.

## Figures and Tables

**Table 1:** Classification of complex DNA damage coupled with BD in the present model.

DNA Damage Type	Symbol	Complexity	Event/Cluster *N*_cl_ *	Model for Yield Calculation
Single-strand breaks	SSB	Isolated	*N*_cl_ < 2	*Y*_SSB_, (Equation (2))
Double-strand breaks (+ base damage)	DSB	Complex	2 ≤ *N*_cl_ < 11	*Y*_DSB_× *f* (2 ≤ *N*_cl_ < 11), (Equation (3))
	DSB/BD	Complex	11 ≤ *N*_cl_ < 20	*Y*_DSB_× *f* (11 ≤ *N*_cl_ < 20), (Equation (3))
	DSB/BD/BD	Complex	20 ≤ *N*_cl_ < 29	*Y*_DSB_× *f* (20 ≤ *N*_cl_ < 29), (Equation (3))
Isolated base damage	BD	Isolated	*N*_cl_ < 2	*Y*_BD_, (Equations (4))
Complex base damage	BD/BD	Complex	2 ≤ *N*_cl_ < 11	*Y*_cBD_× *f* (2 ≤ *N*_cl_ < 11), (Equation (5))
	BD/BD/BD	Complex	11 ≤ *N*_cl_ < 20	*Y*_cBD_× *f* (11 ≤ *N*_cl_ < 20), (Equation (5))
	BD/BD/BD/BD	Complex	20 ≤ *N*_cl_ < 29	*Y*_cBD_× *f* (20 ≤ *N*_cl_ < 29), (Equation (5))

* The maximum inter-lesion distance, *L*_c_, was set to be 10 bp for sampling the events per cluster.

**Table 2:** Classification of complex DSB coupled with strand breaks in the present model.

DNA Damage Type	Symbol	Complexity	Event/Cluster *N*_cl_ *	Model for Yield Calculation
Single-strand breaks	SSB	Isolated	*N*_cl_ < 2	*Y*_SSB_, (Equation (2))
Double-strand breaks (+ strand breaks)	DSB	Simple	2 ≤ *N*_cl_ < 14	*Y*_DSB_ × *f* (2 ≤ *N*_cl_ < 14), (Equation (3))
	DSB+	Complex	14 ≤ *N*_cl_ < 26	*Y*_DSB_ × *f* (14 ≤ *N*_cl_ < 26), (Equation (3))
	DSB++	Complex	26 ≤ *N*_cl_ < 38	*Y*_DSB_ × *f* (26 ≤ *N*_cl_ < 38), (Equation (3))

* The maximum inter-lesion distance, *L*_c_, was set to be 10 bp for sampling the events per cluster.

**Table 3:** Benchmark test for complex double-strand break (DSB) compared to the other simulations.

Electron Energy (keV)	Type of Simulation Code	DNA Strand Break Yields (%)				Yield Ratio (%)
		SSB ^(i)^	DSB ^(ii)^	DSB+ ^(iii)^	DSB++ ^(iii)^	cDSB/DSB ^(iv)^
0.30	PHITS	87.30	10.13	2.57	0.00	20.24
	Geant4-DNA	93.89	4.89	1.22	0.00	20.00
	KURBUC	87.19	9.18	3.28	0.35	28.31
1.00	PHITS	89.60	7.18	3.19	0.04	31.03
	Geant4-DNA	94.62	4.46	0.87	0.05	17.13
	KURBUC	90.65	6.51	2.55	0.29	30.38
10.0	PHITS	93.94	4.62	1.40	0.04	23.81
	KURBUC	96.59	2.61	0.72	0.08	23.45
100	PHITS	94.69	3.71	1.53	0.07	30.18
	KURBUC	96.64	2.69	0.63	0.05	20.07

(i) SSB = single-strand break; (ii) DSB = double-strand break; (iii) DSB+ and DSB++ = DSB coupled with a strand break and two strand breaks within 10 bp, respectively; (iv) cDSB is the sum of the percentages of DSB yields coupled with strand breaks (DSB+ and DSB++).
